# Sowerby's beaked whale biosonar and movement strategy indicate deep-sea foraging niche differentiation in mesoplodont whales

**DOI:** 10.1242/jeb.243728

**Published:** 2022-05-12

**Authors:** Fleur Visser, Machiel G. Oudejans, Onno A. Keller, Peter T. Madsen, Mark Johnson

**Affiliations:** 1 Department of Freshwater and Marine Ecology, IBED, University of Amsterdam, 1090 GE, Amsterdam, The Netherlands; 2 Department of Coastal Systems, NIOZ Royal Netherlands Institute for Sea Research, 1790 AB, Den Burg, Texel, The Netherlands; 3 Kelp Marine Research, 1624 CJ, Hoorn, The Netherlands; 4 Department of Animal Ecology, Utrecht University, 3584 CS, Utrecht, The Netherlands; 5 Zoophysiology, Department of Biology, Aarhus University, DK-8000 Aarhus, Denmark; 6 Aarhus Institute of Advanced Studies, Aarhus University, DK-8000 Aarhus, Denmark

**Keywords:** *Mesoplodon bidens*, Ziphiidae, Niche differentiation, Echolocation, Deep-sea foraging ecology, Pace-of-life syndrome

## Abstract

Closely related species are expected to diverge in foraging strategy, reflecting the evolutionary drive to optimize foraging performance. The most speciose cetacean genus, *Mesoplodon*, comprises beaked whales with little diversity in external morphology or diet, and overlapping distributions. Moreover, the few studied species of beaked whales (Ziphiidae) show very similar foraging styles with slow, energy-conserving movement during long, deep foraging dives. This raises the question of what factors drive their speciation. Using data from animal-attached tags and aerial imagery, we tested the hypothesis that two similar-sized mesoplodonts, Sowerby's (*Mesoplodon bidens*) and Blainville's (*Mesoplodon densirostris*) beaked whales, exploit a similar low-energy niche. We show that, compared with the low-energy strategist Blainville's beaked whale, Sowerby's beaked whale lives in the fast lane. While targeting a similar mesopelagic/bathypelagic foraging zone, they consistently swim and hunt faster, perform shorter deep dives, and echolocate at a faster rate with higher frequency clicks. Further, extensive near-surface travel between deep dives challenges the interpretation of beaked whale shallow inter-foraging dives as a management strategy for decompression sickness. The distinctively higher frequency echolocation clicks do not hold apparent foraging benefits. Instead, we argue that a high-speed foraging style influences dive duration and echolocation behaviour, enabling access to a distinct prey population. Our results demonstrate that beaked whales exploit a broader diversity of deep-sea foraging and energetic niches than hitherto suspected. The marked deviation of Sowerby's beaked whales from the typical ziphiid foraging strategy has potential implications for their response to anthropogenic sounds, which appears to be strongly behaviourally driven in other ziphiids.

## Abstract (precis)

**Summary:** Atypically fast beaked whale in a speciose genus of physically similar whales reveals foraging and energetic niche differentiation in the deep sea.

## INTRODUCTION

The development of echolocation and breath-hold diving capabilities has enabled the radiation of toothed whales into a broad range of marine and aquatic niches, including the deep sea, where they tap into a stable, diverse and abundant fauna (Johnson et al., 2004; Kooyman, 2009; Ramirez-Llodra et al., 2010; Visser et al., 2021a,b). But despite targeting broadly similar fish and squid resources, some cosmopolitan deep-foraging toothed whales have very different diving and foraging styles (Visser et al., 2021a). Pilot whales (*Globicephala* sp.; 5–7 m) exemplify an energetic fast-attacking strategy in which just one large prey may be taken in a mesopelagic deep dive (Aguilar de Soto et al., 2008). In contrast, the much larger body size of sperm whales (*Physeter macrocephalus*; 11–19 m) enables longer dives in which they hunt tens of prey with a relatively slow hunting style (Miller et al., 2004; Watwood et al., 2006). Despite the much smaller body size of most beaked whales (Ziphiidae), the few data available for them indicate a foraging behaviour similar to that of sperm whales. Thus, an energy-conserving movement style appears key to enable the distinctive long and deep foraging dives of these species (Madsen et al., 2014; Tyack et al., 2006).

The 16 species of *Mesoplodon* comprise the most speciose genus within the beaked whales. These small, 4–6 m whales have a highly conserved body plan with a spindle-shaped body and a distinctive rostrum (Carroll et al., 2021; Pitman, 2009). Although detailed information is missing for most species, mesoplodonts are assumed to use echolocation to hunt relatively small fish, squid and crustaceans in the mesopelagic and bathypelagic (Johnson et al., 2004; MacLeod et al., 2003; Mead, 1989). Mesoplodonts are cosmopolitan and multiple species can be found in preferred habitats (Pitman, 2009). For example, all four species known from the North Atlantic, as well as two species of larger non-mesoplodont beaked whales (Cuvier's beaked whale, *Ziphius cavirostris*; and Northern bottlenose whale, *Hyperoodon ampullatus*), are present around the steep oceanic islands of the Azores (Macleod et al., 2005; Silva et al., 2014). Such similar predators with overlapping range are expected to diverge in foraging strategy so as to effectively partition resources, reflecting the evolutionary drive to optimize foraging benefits (Parker and Smith, 1990; Siemers and Schnitzler, 2004). There are indications that niche is structured by size in beaked whales as a whole. The larger Cuvier's beaked whale and Northern bottlenose whale rely primarily on cephalopod prey, whereas the limited diet information on mesoplodonts suggests a preference for fish (MacLeod et al., 2003). However, the lack of differentiation in shape, size and diet within mesoplodonts raises the question of what factors drive their extensive speciation.

As a consequence of their oceanic distribution and inconspicuous surface presence, mesoplodonts are extremely difficult to study and little is known about their behaviour and ecology. An exception is the Blainville's beaked whale (*Mesoplodon densirostris*), found in temperate and tropical waters throughout the world. This species has been the subject of both long-term observations and biologging tagging studies, largely triggered by its involvement, along with Cuvier's beaked whale, in atypical mass strandings associated with navy sonar (reviewed in Hooker et al., 2019).

Similar to larger non-mesoplodont beaked whales, Blainville's beaked whales have a characteristic dive and echolocation pattern in which long (50 min) deep (600–1200 m) foraging dives are interspersed with prolonged intervals of shallow (100–200 m) dives (Tyack et al., 2006). During each deep dive, they produce several thousand echolocation clicks to search for prey, and ∼20–40 rapidly accelerating click sequences, called buzzes, as they attempt to capture prey (Madsen et al., 2013). Their search clicks have a highly distinctive waveform comprising a multi-cycle pulse of 300 µs duration, centred on 35 kHz and with an upwards frequency modulation (FM) from about 25 to 50 kHz (Johnson et al., 2006). Buzz clicks, in comparison, are unmodulated short transients similar to delphinid clicks (Johnson et al., 2006).

Blainville's beaked whales echolocate, and hunt, exclusively in the deeper parts of their 10 or so daily foraging dives, spending only about 20% of the day searching for prey (Arranz et al., 2011). The limited data available from stomach contents and faecal samples suggest a diet mainly composed of a range of small mesopelagic and benthopelagic fish (87%; *N*=132 prey items from 7 individuals), supplemented by cephalopods (9%) and crustaceans (4%) (Allen et al., 2011; Claridge, 2013; Herman et al., 1994; MacLeod et al., 2003; Santos et al., 2007; Sekiguchi et al., 1992). When not performing foraging dives, and during the long low-angle ascents from foraging dives, they are silent (Arranz et al., 2011). These lengthy ascents, and the sequences of shallow non-foraging dives that follow them, have been interpreted as possibly serving to reduce susceptibility to decompression sickness (Bernaldo De Quirós et al., 2019; Hooker et al., 2009), although the mechanism for this has not been identified. Rather, the silent slow ascents during foraging dives, in concert with near-surface acoustic crypsis and a propensity to form small, tight and highly synchronized groups, may serve to avoid predators listening at the surface (Aguilar de Soto et al., 2020). This apparent strong mitigation of predation risk combined with a relatively slow swimming style suggests that the Blainville's beaked whale strategy is to avoid predators rather than out-run them (Aguilar de Soto et al., 2020).

The apparent low-energy lifestyle of Blainville's beaked whales is matched by hypotrophy of many internal organs including the brain, liver and lungs (Pabst et al., 2016). However, paradoxically, Blainville's beaked whales swim muscles have a high proportion of fast-twitch fibres that are more commonly found in sprinting animals. These fibres may serve as an oxygen store for neighbouring slow-twitch fibres during long foraging dives (Velten et al., 2013), but may also be used to power their low-angle ascents, in which a distinctive stroke-and-glide gait with energetic fast strokes is employed (Martín López et al., 2015). Taken together, these findings on Blainville's beaked whales paint a picture of a specialized low-energy mammalian predator that has shaped its metabolic rate and behaviour to suit a remote, deep-sea foraging niche.

Data on other mesoplodonts are largely limited to rare observations (e.g. Aguilar de Soto et al., 2017), and acoustic recordings of their echolocation signals. Mesoplodont species for which data are available produce mid-frequency (30–100 kHz) FM clicks that may be distinct enough to distinguish some species (Baumann-Pickering et al., 2013; DeAngelis et al., 2018). However, it is unclear whether this frequency diversity relates to different foraging niches. Although niche may shape some aspects of sound production in toothed whales (Jensen et al., 2018), predation risk may also be a strong driver (Madsen et al., 2005; Morisaka and Connor, 2007). Acoustic divergence can also help to maintain group identity in sympatric species, as may be the case for some bats (Chaverri et al., 2018) and delphinids (Kyhn et al., 2010). Thus, despite the diversity in echolocation clicks and overlapping range of mesoplodonts (Macleod, 2000), the similarity in size, shape and diet leads us to predict a uniformity of niche across this genus.

Here, we report the first biologging data from Sowerby's beaked whale (*Mesoplodon bidens*), a relatively abundant mesoplodont, found in the North Atlantic north of about 30° latitude. Sowerby's beaked whales and Blainville's beaked whales are very similar in size (5–6 m), and are partially sympatric in the North Atlantic with an overlapping southerly/northerly distribution range. Like Blainville's, Sowerby's beaked whales feed mostly on small mesopelagic and bathypelagic fish (97.6%; *N*=10,173 prey items from 31 individuals), including on the main prey families reported from the diet of Blainville's beaked whales (e.g. Myctophidae, Gadidae) (MacLeod et al., 2003; Pereira et al., 2011; Santos et al., 2007; Spitz et al., 2011; Wenzel et al., 2013). There are conflicting reports that Sowerby's beaked whales produce echolocation clicks that are either distinctly lower or higher in frequency than those of Blainville's beaked whales (Cholewiak et al., 2013), but behavioural data are completely absent. We investigated the swimming and echolocation behaviour of Sowerby's and Blainville's beaked whales to test the null hypothesis that mesoplodonts with a comparable bauplan exploit a similar low-energy niche.

## MATERIALS AND METHODS

### Tag and aerial data recording

Two Sowerby's beaked whales (*Mesoplodon bidens* Sowerby 1804) in groups of 3 and 5 individuals were tagged off Terceira Island, Azores, in the summer of 2017 and 2018 (respectively mb17 and mb18, hereafter). Suction-cup-attached sound and movement tags (DTAG v4; Johnson and Tyack, 2003) were deployed on animals using an 8 m hand-held pole, from a 6.2 m rigid-hulled inflatable boat. The tags were programmed to detach after 4 and 6 h because the unknown movement patterns of the animals could result in longer-lasting tags detaching far from shore. Tagged whales were not followed, in an effort to minimize observer pressure on the animals. However, tags were monitored from a distance using VHF radio tracking and were recovered once detached. The tags recorded sound (175 dB re. 1 µPa clip level, 0.2–160 kHz bandwidth, 16-bit resolution, 576 kHz sampling rate), synchronously with motion and orientation data from a depth sensor (50 Hz sampling rate) and triaxial accelerometers (250 Hz sampling) and magnetometers (50 Hz sampling). In addition, the near-surface behaviour of nine groups, each comprising 2–6 non-tagged individuals, was recorded using aerial video recordings by an unpiloted autonomous system (UAS; DJI Phantom 4 Pro). Aerial recordings were made by positioning the UAS over the sighting location of a group of Sowerby's beaked whales and tracking the group for as long as they were visible between deeper dives. When the limited UAS battery capacity (∼20 min) required early return to the research vessel, an attempt was made to relocate the group following battery switch.

### Animal welfare

All research was conducted under scientific permits issued by the Direção Regional dos Assuntos do Mar, Secretaria Regional do Mar, Ciência e Tecnologia, Horta, Faial, Azores, Portugal.

### Movement analysis

Motion data from the tag accelerometers and magnetometers were examined to identify stroke rate, gait and swimming speed. The sensor data were first calibrated and corrected for the approximate orientation of the tag on the whales using standard methods (Johnson and Tyack, 2003) and were then decimated to a common sampling rate of 25 Hz. Stroke rate is expected to be linearly related to swim speed, and animals of similar size and shape, with similar swimming styles, should obtain a similar speed when stroking at about the same rate (Wardle, 1975). To detect individual locomotory strokes, the dominant stroke frequency (DSF) (*sensu* Sato et al., 2007) was first estimated for each animal. The DSF was taken as the peak frequency in the spectral average (256-point FFT, 50% overlap) of the dorso-ventral accelerometer signal, discounting peaks <0.1 Hz that result from orientation changes. Body rotations associated with stroking were then estimated from the triaxial magnetometer signals using the method of Martín López et al. (2015). Briefly, the magnetometer signals were divided into low-frequency components related to orientation changes, and higher frequency components related to locomotion, using complementary low-pass and high-pass filters with a cut-off frequency of one-half of the DSF. These and all other filtering operations were performed using linear-phase delay-corrected symmetric finite impulse response (FIR) filters. The body rotation signal was then computed from the low-pass and high-pass filtered components following eqns A6 and A7 in Martín López et al. (2015). Individual half-strokes were detected in the body rotation signal using a hysteretic zero-crossing detector with a threshold of 2 deg. The stroke interval was taken as the time between successive positive-going zero crossings, with intervals longer than 2/DSF (i.e. glides) excluded. In both whales, the stroking rate (i.e. the inverse of the stroke interval) was clearly elevated in the first 30 min after tagging and this interval was excluded from analyses of mean rate.

For gait analysis, the root-mean-squared (RMS) heave acceleration for each stroke interval was calculated by first high-pass filtering the dorso-ventral axis of the triaxial accelerometer signals with the same high-pass filter as used with the magnetometer signals. The filtered acceleration samples corresponding to each stroke interval were then identified and the RMS computed. Histograms of the RMS heave acceleration for each whale were bimodal with a subset of strokes having distinctly higher acceleration. The break-point between the two modes in the histogram for each whale was used as a threshold to separate regular and high-acceleration strokes.

The sensor data were decimated to a second lower sampling rate of 5 Hz for estimation of orientation (pitch, roll and heading) and speed. Forward speed, i.e. the speed in the direction of motion, was estimated by first computing the change in depth over time using the pressure sensor. A 0.3 Hz low-pass filter was applied to attenuate fluctuations in this vertical speed due to individual stroke cycles. The forward speed was then estimated as the filtered vertical speed, divided by the sine of the pitch angle (Miller et al., 2004), where the same low-pass filter was applied to the acceleration prior to computation of pitch angle. Speed estimates with an absolute pitch angle below 20 deg were removed from analysis to avoid amplifying errors in the vertical speed (Miller et al., 2004). For descent and ascent speed analysis, dives were divided into descent, search and ascent phases. The descent phase extends from when the whale leaves the surface to the start of regular clicking, while the ascent phase is the interval from the end of regular clicking until the animal re-gains the surface. The search phase is the time between descent and ascent.

To characterize behaviour between deep dives, aerial video recordings of groups were analysed for near-surface presence and swimming speed. An operational definition of near-surface was used, defined as visible in the UAS video recording (relating to an estimated maximum depth of ∼20 m). Video recordings were annotated to identify the timing of surfacings of each individual in the group and the timings were supplemented by visual observations of the group when the UAS returned for a battery change. The resulting tracking durations were used to identify the minimum duration of continuous near-surface presence (as it was rarely possible to capture the precise time when animals first returned to the surface from a deep dive). Travel speed was estimated in periods when the UAS was directly overhead of at least one individual in the group, so that UAS speed reflected individual swimming speed.

### Acoustic analysis

The tags recorded sounds from the tagged whale as well as from nearby conspecifics, and other animals in the vicinity. Sound data were examined 15 s at a time using a spectrogram display (256-point FFT, 50% overlap) and selective listening to identify the timing of echolocation buzzes, the start and end of regular clicking in dives, and other sounds. Shore- and vessel-based visual observers did not record other species of beaked whales on the days of the tag deployments. Therefore, all high signal-to-noise ratio (SNR) FM clicks recorded on the tags, and all buzzes following FM click trains were classified as originating from Sowerby's beaked whales. A supervised click detector was used to determine the precise timing of all FM search and buzz clicks. This was run twice with different settings on each recording, first to detect the clicks from the tagged whale and then to detect the clicks from other nearby animals. As for clicks from other odontocetes, clicks from the tagged whales were readily distinguishable from those of other animals by the presence of a low-frequency component in the former (Johnson et al., 2009; Zimmer et al., 2005). Based on spectrogram evaluation, a low-frequency (5–30 kHz) bandpass filter was used to detect clicks from the tagged whale, while a 30–120 kHz filter was used to detect clicks from other animals. Click detections were checked, and amended when necessary, by comparison of detection times with the corresponding spectrograms and Hilbert envelopes. The inter-click interval (ICI), indicative of the maximum unambiguous echolocation inspection range (ICI×sound speed in water/2; e.g. Jensen et al., 2018), was recorded for all click trains.

Buzzes were distinguished from regular clicks by the rapidly accelerating click rates in the former (Johnson et al., 2006). A threshold of 50 ms was used to define the start and end of buzzes based on a visual examination of ICI patterns but, given the rapidly changing click rate in buzzes, a lower or higher threshold would yield similar buzz durations. Individual buzz clicks were detectable in the recording from mb17 but not mb18 because of a more posterior tag position on the body. These were used to construct echograms with time–range axes following the method of Johnson (2014). Targeted prey appear in these echograms as a sequence of echoes with progressively shorter range during the buzz. Often, echoes from the same target are visible during slower clicking prior to the buzz, allowing estimation of the range to the prey at the start of the buzz (the so-called hand-off distance; Johnson et al., 2006). Likewise, the closing speed on targeted prey can be inferred from the slope of the echo trace in the echogram. Elusive behaviour of prey is evidenced by sudden changes in the slope due to rapid swimming of the prey (Vance et al., 2021). In all buzzes for which prey movement was evident in the echogram, the closing speed was measured prior to the first detectable response of the prey.

The waveform and spectrum of echolocation clicks cannot be inferred reliably from the clicks of the tagged animal as the tag location is behind the head and therefore far from the centre of the forward-directed beam (Johnson et al., 2009). However, sequences of high-intensity clicks were frequently recorded during foraging dives that were not produced by the tagged whale. Given the depth at which these were recorded, it is reasonable to assume that they were produced by other Sowerby's beaked whales swimming near the tagged whales. Click intensity generally increased and then decreased within each sequence as the clicking conspecific whale changed orientation, sweeping its forward-directed biosonar beam past the tag. We defined sequences of clicks from untagged whales as comprising 3 or more clicks, starting and ending when the ICI was more than twice the average ICI of the tagged whales, i.e. implying a missing click. To pick a sub-set of clicks from these sequences that best represented the on-axis click waveform, we applied the following criteria: (i) sequences must have a difference in intensity of at least 10 dB between the weakest and strongest click, and (ii) the strongest click in the sequence must not be the first or last click in a sequence and must not be clipped in the recording. The strongest click in each such sequence was extracted for analysis after bandpass filtering (10–200 kHz) to reduce extraneous noise. Temporal and spectral parameters for each potential on-axis click were calculated following Johnson et al. (2006). The waveforms were checked for consistency with those of occasional strong echoes from tagged whale clicks. Buzz clicks were recorded often from untagged whales but generally had low SNR consistent with the typically lower source level of these clicks in odontocetes (Madsen et al., 2005; Wisniewska et al., 2014). Exemplar clicks with SNR of >20 dB were hand-selected from buzzes to give a rough idea of the click parameters. All analyses were performed in Matlab (The MathWorks Inc.) using custom functions available at www.animaltags.org and www.soundtags.org.

## RESULTS

### Foraging dives

**Fig. 1.**
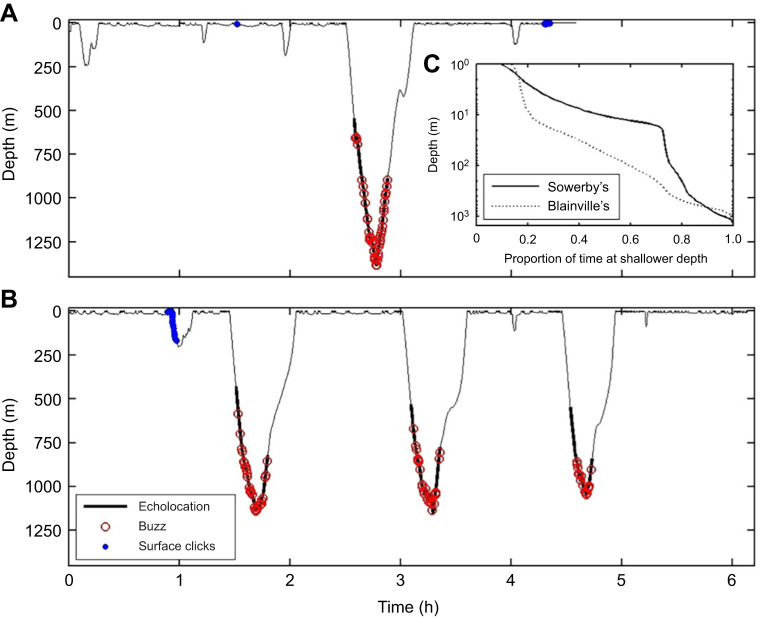
**Dive and acoustic behaviour of two Sowerby's beaked whales (*Mesoplodon bidens*).** Individuals (A) mb17 (mb17_224a) and (B) mb18 (mb18_219a) were tagged off Terceira Island, Azores, with DTAG sound and movement tags (mb17: 14:00–18:33 h local time, 12 August 2017; mb18: 12:46–18:55 h, 7 August 2018). Thick traces indicate where regular echolocation clicks were produced and delimit the search phase of foraging dives. Buzzes (presumed prey capture attempts) are shown by red circles. Blue dots near the surface indicate recordings of bouts of clicks from other toothed whales of uncertain species. (C) Cumulative histogram showing the proportion of time spent at depths shallower than those on the vertical axis by the two Sowerby's beaked whales and six Blainville's beaked whales (*Mesoplodon densirostris*; 107 h, from El Hierro, Canary Islands; data courtesy of N. Aguilar).

During the 4 and 6 h tag attachments, mb17 and mb18 performed one and three V-shaped deep dives, respectively (Fig. 1, Table 1). All four dives contained regular echolocation clicks and multiple buzzes, strongly indicating a foraging function. Echolocation, and therefore the search for prey, started at 400–550 m depth on the descent and ended early in the ascent, at depths of 770–880 m (mean±s.d. depth of clicking: 927±157 m). Although the foraging dives were comparably deep as those of other beaked whales (Tyack et al., 2006), they were distinctly shorter (29–37 min), leading to 12–18 min search times with echolocation (Table 1). The whales produced short buzzes (median duration: 1.8 s, interquartile range IQR: 0.9 s, range: 0.5–6 s) in quick succession (median inter-buzz interval: 20 s). Prey were targeted across a broad depth range (buzz depths: 670–1386 m; mean±s.d.: 967±120 m). The first buzz in each dive occurred 50–224 s after and 109–350 m deeper than the start of clicking. Assuming that buzzes indicate prey capture attempts on individual prey, as in other odontocetes (Wisniewska et al., 2014), the combination of brief and closely spaced buzzes enabled the whales to target 17–42 prey per dive, similar to Blainville's beaked whales, despite the shorter search time (Table 1).

**Table 1.**
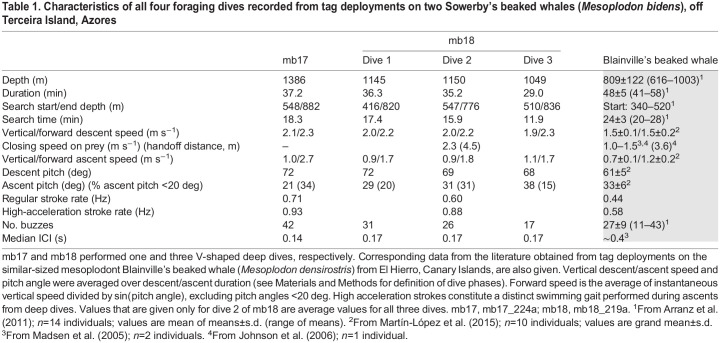
Characteristics of all four foraging dives recorded from tag deployments on two Sowerby's beaked whales (*Mesoplodon bidens*), off Terceira Island, Azores

### Deep-dive locomotion strategy

Descents in deep dives were steep and involved extensive gliding, indicating a net tissue density greater than that of seawater (Miller et al., 2004). Descent speeds were therefore constrained by the terminal glide speed (Biuw et al., 2003), which was about 2 m s^−1^ for both animals (Table 1). As with Blainville's and Cuvier's beaked whales, the ascents were silent and were performed at low pitch angles (20–40 deg), leading to them accounting for about 40% of the duration of the deep dives (Fig. 1). Ascents were performed with a distinctive stroke-and-glide gait which has only been reported for beaked whales ascending from deep dives (Martín López et al., 2015). This comprises bursts of regular stroking inter-mixed with shorter high-acceleration strokes and brief glides. Both types of stroke, however, were substantially faster for Sowerby's beaked whales than for Blainville's beaked whales (Table 1) (Martín López et al., 2015). The faster stroking rates in Sowerby's beaked whales were also associated with a much higher average forward speed during ascents, estimated at 2.7 and 1.7 m s^−1^ for mb17 and mb18, respectively (Table 1).

### Sound production

**Fig. 2.**
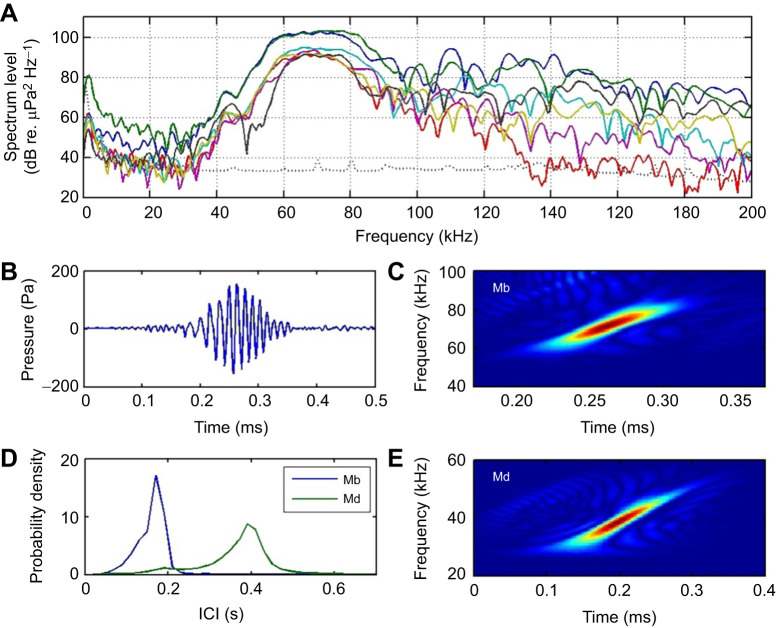
**Characteristics of Sowerby's beaked whale echolocation clicks.** (A) Spectra of six exemplar frequency-modulated (FM) search clicks. These clicks were recorded from individuals swimming near a tagged whale and were judged to be recorded close to the centre of the directional sonar beam (near on-axis). The dotted line indicates the noise floor of the recording, combining ambient and system noise. (B) Waveform of one of these clicks. (C) Time–frequency (Wigner) plot of the same click. (D) Inter-click interval (ICI) probability density function estimates for Sowerby's (Mb) and Blainville's beaked whale (Md), demonstrating the widely differing typical click rates. Sowerby's beaked whale curve was produced from 20,330 clicks recorded from two individuals. Blainville's beaked whale curve was produced from 80,900 clicks recorded from four individuals (courtesy of N. Aguilar). (E) Time–frequency plot of a representative on-axis Blainville's beaked whale click (note the different time and frequency scale compared with C).

Tagged Sowerby's beaked whales produced echolocation clicks at a relatively constant ICI of 0.14–0.17 s throughout the search phase of dives. This ICI results in a click rate that is 2–3 times higher than that recorded for Blainville's beaked whales (Table 1, Fig. 2; Madsen et al., 2005; Zimmer et al., 2005). Clicks from multiple untagged whales were detectable almost continuously throughout the search phases of all four deep dives. Candidate on-axis clicks, with received level greater than 155 dB re. 1 µPa peak–peak and excellent SNR, were analysed from 52 click sequences (Table 2). The clicks were all multi-cycle, Gaussian-shaped pulses with upwards FM (Fig. 2). They are therefore very similar in form to the distinctive FM clicks of Blainville's beaked whales. However, the centroid frequency of the Sowerby's beaked whale FM clicks, at 72 kHz, is almost twice that of Blainville's beaked whales. The FM also extends over a wider range than for Blainville's beaked whales, resulting in a bandwidth that is ∼20–40% higher. But the Sowerby's beaked whale clicks are distinctly shorter than those of Blainville's beaked whales, resulting in a very similar time–bandwidth product. The combination of short duration and high modulation range results in a FM rate that is twice as high in Sowerby's beaked whale clicks, compared with those of Blainville's beaked whales (Table 2).

**Table 2.**
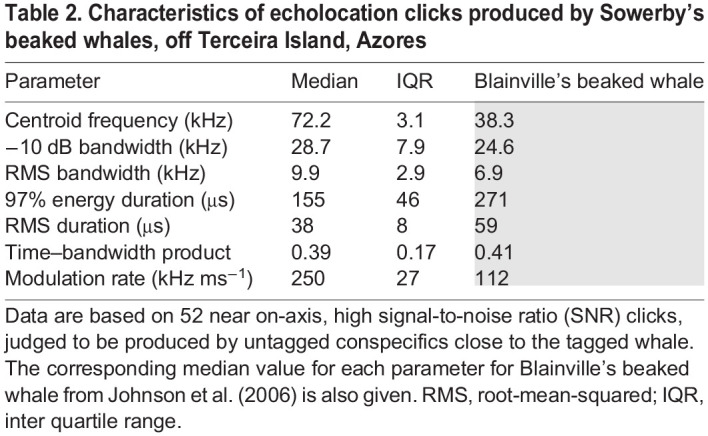
Characteristics of echolocation clicks produced by Sowerby's beaked whales, off Terceira Island, Azores

**Fig. 3.**
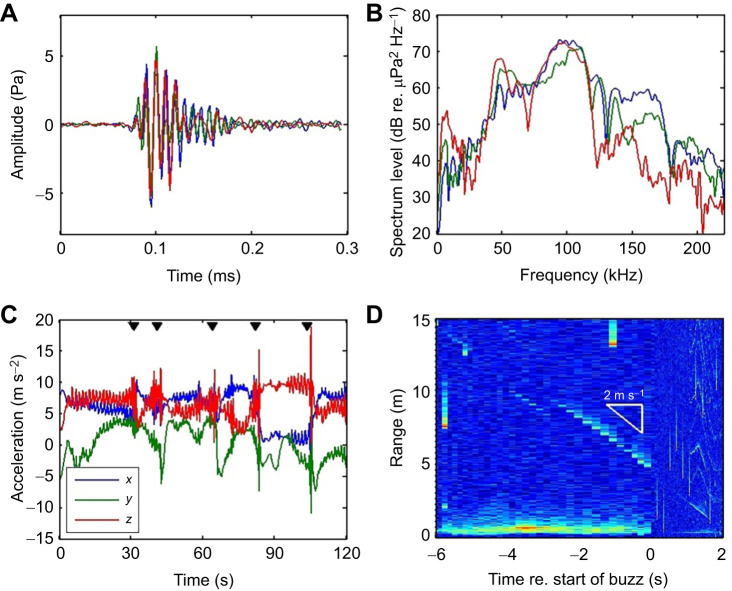
**Echolocation and movement during prey capture attempts in Sowerby's beaked whale.** (A) Waveforms of three buzz clicks recorded from untagged whales near a tagged whale (denoted by different colours). (B) Spectral density of the same three clicks. Variability in the spectra suggests that these clicks were probably not recorded close to the biosonar acoustic axis (off-axis). (C) Accelerometer data recorded during a sequence of buzzes produced by the tagged whale, at about 1020 m depth. The start times of buzzes are indicated by the black triangles. Sharp transients occur in the *x* and *z* accelerometer axes during the buzzes, and rapid cyclic variations in the same axes prior to each buzz indicate energetic swimming. (D) Echogram during a typical buzz (the 5th buzz in C) showing a steady approach speed of nearly 2 m s^−1^. The target is first visible in the echogram 6 s before the start of the buzz at a range of 15 m. The buzz begins when the target is at about 5 m range and there is no indication of an escape response by the targeted organism.

The tags recorded buzzes from nearby untagged whales throughout deep dives (Fig. 3). The occurrence of these at great depths and amidst FM search clicks makes it highly likely that these are produced by Sowerby's beaked whales. The variability in the waveform and spectra of the buzz clicks suggests that none, or few, of these were recorded close to the biosonar beam axis. The observed buzz clicks were unmodulated transients, as also seen for Blainville's beaked whales (Johnson et al., 2008). However, the Sowerby's beaked whale buzz clicks had a very high centroid frequency of 90–100 kHz, a 20 kHz RMS bandwidth and a RMS duration of about 30 µs (Fig. 3). Sowerby's beaked whale buzz clicks were therefore distinctly higher in frequency and shorter than their own FM search clicks, and also buzz clicks produced by Blainville's beaked whales.

### Prey characteristics

Buzzes occurred over a wide depth range (190–730 m depth difference between the shallowest and deepest buzz within a dive), indicating that prey were very broadly distributed with depth. Individual buzz clicks from the tagged whales were only reliably detected in mb17. Echograms produced for these buzzes showed prey targets in about 75% of cases although prey behaviour could be inferred in only a subset of these. Prey were elusive (i.e. attempted to escape) in 17 of the 29 buzzes for which a behavioural determination could be made. However, escapes only appeared to be successful in two buzzes, indicating a capture rate of 93%, albeit based on a very small sample. Both tagged Sowerby's beaked whales swam actively prior to most buzzes (Fig. 3), resulting in typically high closing speeds on prey (median 2.3 m s^−1^, IQR 0.6, range 1.2–3.1 m s^−1^, *n*=32), twice the prey approach speed reported for Blainville's beaked whales (Table 1). Prey were relatively far from the whales at the start of buzzes (median 4.5 m; Table 1) and these so-called handoff distances were correlated with closing speed (slope 1.2 m per m s^−1^, *r*^2^=0.44, *n*=27 buzzes from one animal).

### Behaviour between foraging dives

Tagged Sowerby's beaked whales spent lengthy intervals close to the surface after each deep dive (Fig. 1). The resulting dive cycle duration (i.e. the time between the start of successive deep dives) was 94 and 87 min (*n*=2 intervals, both for mb18). Blainville's beaked whales, and all other ziphiids recorded thus far, typically perform a sequence of relatively long shallow (20–240 m) dives between deep foraging dives, especially during daylight hours (Baird et al., 2006; Tyack et al., 2006). The two Sowerby's beaked whales, however, performed very few shallow dives (3–4 dives, to depths of 90–240 m), that accounted for only 7–14% of the time recorded outside deep dives. The vast majority of the time between foraging dives was spent either breathing at the surface or in short (median 40 s) shallow (<20 m) submergences. Whales stroked almost continuously during these submergences, suggesting a travelling behaviour (see Movie 1 for example). For the two tagged whales, median stroke rates between dives were similar to those during deep dives (0.76 and 0.60 Hz for mb17 and mb18, respectively). However, in the two complete inter-deep-dive intervals of mb18 (i.e. intervals that start and end with a deep dive), stroking rates were significantly higher in the first 10 min after each deep dive than in the remainder of the interval (median of 0.78 versus 0.57 Hz, respectively, rank sum *P*≈0, *n*=100 strokes selected randomly after each dive within the first 10 min and an equal number in the remaining inter-deep-dive interval). No intervals of logging were recorded. Prolonged near-surface presence and travel were confirmed by aerial video recordings of nine other groups that could be visually tracked at or near the surface for periods of 5–59 min (Table 3; Movie 1). Groups, composed of 2–6 closely spaced individuals, showed synchronous surface movement patterns, with an overall mean near-surface swimming speed of 2.4 m s^−1^ (range: 1.7–3.1 m s^−1^).

**Table 3.**
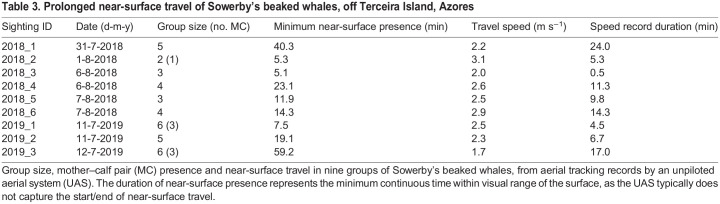
Prolonged near-surface travel of Sowerby's beaked whales, off Terceira Island, Azores

### Interactions

**Fig. 4.**
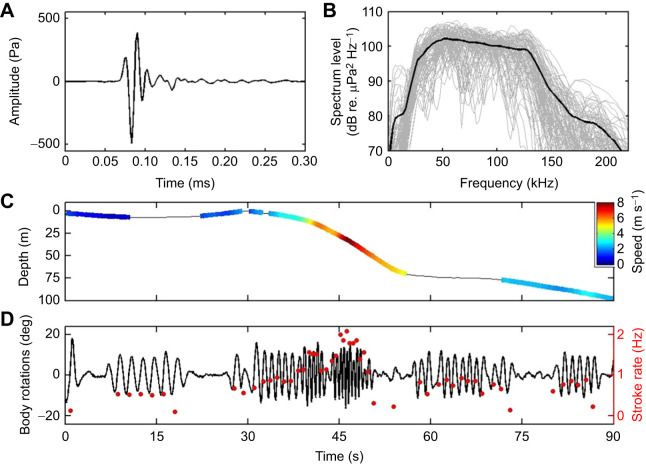
**Nearby vocal animals and high-speed swimming at the surface by Sowerby's beaked whales.** (A) Waveform and (B) spectra of high-intensity clicks recorded near the surface, produced by untagged conspecifics or by toothed whales of another species (blue dots in Fig. 1 show the occurrence of these clicks in the dive profiles of the tagged whales). (C,D) Tagged Sowerby's beaked whales produced occasional bursts of high speed near the surface, reaching estimated forward speeds of 8 m s^−1^ with stroking rates of up to 2 Hz. (C) Dive profile during 90 s of surface swimming coloured by estimated forward speed. Intervals with absolute pitch angle <20 deg when the speed cannot be accurately estimated are uncoloured. (D) Pitching rotations due to swimming as measured at the tag position on the body over the same interval (black line), and inferred instantaneous stroking rate (red dots).

Both tagged whales performed several short bursts of very fast swimming with stroking rates reaching 1.5–2 Hz and estimated forward speeds of 5–8 m s^−1^ (Fig. 4). These bursts all occurred near the surface and were directed downward with a pitch angle of 30–60 deg. One such burst occurred during a bout of surface clicking. Although the tagged whales were silent between foraging dives, short bouts of clicking were recorded by the tags on three occasions when the tagged whales were near the surface (twice on mb17, once on mb18, at depths of 0–170 m). These bouts comprised 500–1500 clicks of varying intensity, with some being strong enough to clip in the tag recording (i.e. >180 dB re. 1 µPa peak–peak). The strongest unclipped clicks in these bouts (*n*=99) were short transients with 45 µs median 97% energy duration, 78 kHz centroid frequency and 27 kHz RMS bandwidth (Fig. 4). Judging by their varying level and ICI, the clicks were produced by multiple animals during each bout. None of the clicks had the low-frequency click component that is normally present in clicks made by the tagged whale. When short sequences of clicks apparently from a single individual could be identified, the ICI was 0.1–0.2 s but there were also some fast sequences of clicks similar to echolocation buzzes. The bouts lasted between 0.5 and 5 min. Several faint whistles were also recorded during two of the bouts. The whistles were mostly simple 0.3–1.2 s up or down sweeps extending from 5 to 20 kHz.

## DISCUSSION

Slow, energy-conserving movement during deep foraging dives, interspersed with shallower, non-foraging dives is a thus far unifying characteristic amongst tagged beaked whales (Ziphiidae), across a large range of body sizes (4–12 m; Miller et al., 2015; Stimpert et al., 2014; Tyack et al., 2006). This diving strategy is thought to help enable extremely long and deep dives into the bathypelagic (Tyack et al., 2006). The apparent lack of diversity in the external morphology of the speciose *Mesoplodon* genus of beaked whales, together with their presumed shared mesopelagic diet, led us to hypothesize that they share the same low-energy niche. However, we show here that similar-sized mesoplodonts with similar diets and partially overlapping distributions, Sowerby's and Blainville's beaked whales (*M. bidens* and *M. densirostris*), differ widely in their sensory and behavioural ecology. Despite being only slightly larger than the slow-moving Blainville's beaked whale (Arranz et al., 2011), Sowerby's beaked whales swim and hunt faster, powered by much more rapid swimming strokes, and perform shorter foraging dives than Blainville's beaked whales – while reaching comparable depths. Whereas breath-hold divers are expected to move conservatively so as to maximize dive duration, tagged Sowerby's beaked whales swam at high speeds even at depths >1000 m, while attempting to capture multiple prey in rapid succession. During these dives, they produce echolocation clicks that have 2 times higher centre frequencies and are produced at twice the rate of those of Blainville's beaked whale clicks. Between foraging dives, tagged Sowerby's beaked whales engaged in prolonged periods of energetic near-surface swimming that contrast strongly with the sequences of shallow dives that have been considered a resting or recovery behaviour in Blainville's beaked whales.

These findings for Sowerby's beaked whales come from only two tagged animals and nine groups tracked using aerial imagery. The use of clicks from other whales swimming near the tagged whales expands the number of animals contributing to the definition of the biosonar signals, but the data size is nevertheless small and the resulting inferences must be treated with appropriate caution. Like most species of beaked whales, Sowerby's beaked whales are extremely difficult to find and approach for tagging. However, the stereotypical nature of both the diving and vocal behaviour in the available data, and the consistent contrast with any similar-sized subset of the more extensive data from Blainville's beaked whales, suggest that some robust points of difference can be established.

### High-frequency echolocation

The centre frequency of Sowerby's beaked whale clicks is unusually high for an animal of this size and goes against the usual pattern of reducing frequency with body size in toothed whales (Jensen et al., 2018). One implication of a higher centre frequency for a given body size is that the biosonar beamwidth will be narrower unless the radiating surface (i.e. the distal end of the melon) has a reduced size. Jensen et al. (2018) showed that beamwidth is broadly conserved across odontocetes and argue that there are strong foraging benefits in a narrow, but not excessively narrow, biosonar beam. As the maxillae of Sowerby's beaked whales are broadly similar in shape and size to those of Blainville's beaked whales, we therefore predict that the soft tissue structures, specifically the melon width, are reduced in size in order to conserve the approximately 10–15 deg half-power beamwidth measured in Blainville's beaked whales (Shaffer et al., 2013) – a hypothesis that warrants future testing.

Despite the 2-fold increase in centre frequency, the echolocation clicks of Sowerby's beaked whales are remarkably similar to those of Blainville's beaked whales, to the extent that a time-dilated version of the former is a close match to the latter. The distinctive linear upwards FM, Gaussian envelope and relatively long duration in both species reinforce the notion that these are ancestral traits. However, the wide difference in centre frequency between the species suggests either a greater selection pressure on this parameter than on the FM characteristic, or conversely a greater pressure to conserve the modulation. In either case, our findings indicate that whatever anatomical structure produces the FM characteristic, it must scale with frequency. The extensive suite of anatomical adjustments presumably needed to accommodate the changes in centre frequency suggests that there are strong behavioural and/or ecological benefits to the particular frequencies manifested by both mesoplodonts.

On the face of it, production of echolocation clicks with a high centre frequency, and therefore greater sound absorption, seems like a poor choice for a deep-diving predator that would benefit from long-range patch assessment (Malinka et al., 2021). Unlike sperm whales (*Physeter macrocephalus*) but similar to Blainville's beaked whales, Sowerby's beaked whales begin clicking when they are already deep in the foraging dive and make their first prey capture attempt relatively soon thereafter. This suggests that they do not make use of extreme long-range echolocation. This notion is further supported by their short and relatively constant ICIs, which have an unambiguous inspection range of ∼105–130 m (i.e. ICI×sound speed/2).

In comparison, Blainville's beaked whales, with an ICI of 0.4 s, have a much larger maximum inspection range of ∼300 m. At this range, the total transmission loss (TL=40log10*r*+2α*r*, where *r* is range in m and α is the absorption in dB per m; Kinsler et al., 1963) is about 104 dB at the centroid frequency of Blainville's beaked whale regular clicks. Because of the increased attenuation of their higher frequency clicks, Sowerby's beaked whales would experience the same transmission loss at a range of about 230 m. Thus, assuming that both species produce clicks with about the same source level, have the same auditory detection threshold and are targeting prey with a similar target strength, Sowerby's beaked whales should be able to detect prey at ranges well beyond the inspection range inferred from their ICI. Hence, by electing to use short ICIs, Sowerby's beaked whales do not seem to exploit the prey detection range afforded by their clicks. This suggests that the higher click frequency of Sowerby's beaked whales does not strongly constrain their behaviourally inferred echolocation range.

A potential benefit of a higher click frequency is that stronger echoes will be returned from small targets. However, the wavelengths of both species' clicks (21 and 40 mm, respectively, for Sowerby's and Blainville's) are short compared with probable prey sizes (∼100–200 mm; MacLeod et al., 2003; Wenzel et al., 2013). Thus, there is probably little difference in echo strength from most prey (Madsen et al., 2007). Moreover, the relatively short duration of beaked whale clicks makes them highly insensitive to Doppler shifts (which might provide information about prey movement), irrespective of centre frequency (Johnson et al., 2006). We conclude therefore that the higher frequencies of Sowerby's beaked whale clicks, relative to those of Blainville's beaked whales, do not appear to confer direct benefits in terms of foraging ecology. This raises the question of what other factors may drive this differentiation.

### Species-specific echolocation signals

An important benefit of readily distinguishable acoustic signals for sympatric species is in species recognition (Kyhn et al., 2010). Sowerby's beaked whales, like other beaked whales, often aggregate in small, possibly ephemeral groups and are observed to dive synchronously. If Sowerby's beaked whales use the echolocation clicks of group members to coordinate foraging dives and to facilitate reunion at the surface, as has been proposed for other beaked whales (Aguilar de Soto et al., 2020), their distinct echolocation click frequencies reduce the possibility of mistaking other species for group members. The click frequencies of other beaked whale species that overlap in distribution range with those of Sowerby's beaked whales and for which high-quality on-axis field recordings are available, Gervais' (*Mesoplodon europaeus*), True's (*Mesoplodon mirus*) and Cuvier's beaked whale (*Z. cavirostris*) and Northern bottlenose whale (*H. ampullatus*), cover the 20–55 kHz frequency range (DeAngelis et al., 2018; Gillespie et al., 2009; Wahlberg et al., 2011; Zimmer et al., 2005), well below the 60–85 kHz for Sowerby's beaked whale clicks. This benefit also extends to passive acoustic monitoring (PAM) studies where reliable classification of species is fundamental for accurate density and distribution estimates of vocal species (Marques et al., 2013). The secure characterization of Sowerby's beaked whale echolocation signals enables re-interpretation of existing PAM recordings (such as Cholewiak et al., 2013), provided that these have sufficiently high sampling rate (i.e. 192 kHz or higher). The Sowerby's beaked whale clicks reported here are similar to clicks of an unidentified beaked whale species ‘BW70’, recorded in the Gulf of California (Baumann-Pickering et al., 2013). As Sowerby's beaked whales have only been reported in the North Atlantic (Macleod, 2000), the use of similar high-frequency clicks across oceans perhaps suggests a convergence of acoustic niches between different beaked whale species.

### Reduced detectability

Although sound attenuation associated with the high-frequency Sowerby's beaked whale clicks appears to have little impact on foraging (i.e. because of the short ICI), it considerably reduces the detection range of these signals by eavesdropping predators, competitors and PAM systems. In extremely quiet conditions, Blainville's beaked whale clicks can be detected at ranges up to 6 km (Shaffer et al., 2013), at which the attenuation from spreading and absorption are roughly 75 and 40 dB, respectively. Assuming that Sowerby's and Blainville's beaked whales produce clicks with similar on-axis source levels and using the same total attenuation (115 dB) to set an upper limit on detectability, Sowerby's beaked whale clicks would be detectable at less than 2.4 km (68 dB spreading and 48 dB absorption), resulting in 16% [i.e. (2.4/6)^2^] of the detection area compared with that for Blainville's beaked whales. However, under more typical elevated ambient noise conditions, this difference may be less pronounced.

### The fast lane

Aside from the centre frequency of clicks, Sowerby's beaked whale foraging behaviour differs in three key respects from that of Blainville's beaked whales: they swim and hunt faster, the dives are shorter and they use higher click rates (6–7 versus 3 per second for Blainville's beaked whales). This begs the question of whether these characteristics are interrelated and form a strategy targeted at a particular prey type. Both tagged Sowerby's beaked whales performed consistently fast prey attacks (inferred from stroke rate in mb17; 2.3 m s^−1^ measured in mb18) and short buzzes, suggesting an energetic foraging style in which prey are overcome quickly, leaving little room for escape attempts. The high speeds are a result of locomotor strokes which are ∼50% faster than those of the slightly smaller Blainville's beaked whales. Both tagged Sowerby's beaked whales targeted prey resources over a broad depth range, in V-shaped dives that are similar to those of pilot whales and Risso's dolphins (*Grampus griseus*), both high-speed mesopelagic foragers (Aguilar de Soto et al., 2008; Visser et al., 2021b). This hints at selective foraging on a broadly distributed energetic fauna. Although Sowerby's and Blainville's beaked whales both appear to primarily target mesopelagic fish (MacLeod et al., 2003; Santos et al., 2001, 2007), the more energetic foraging style of the former could indicate a niche comprising the larger and/or more muscular individuals within a prey community, or supported by the relatively nutrient-rich high-latitude waters preferred by Sowerby's beaked whales.

Irrespective of diet, however, such a high-speed foraging style is predicted to involve higher basal and active metabolic costs which may contribute to the shorter dive durations. Likewise, the higher sonar sampling rate could be matched to the increased swimming speed, providing a similar information flow per metre covered to that for the slower swimming Blainville's beaked whales (Madsen et al., 2013). We therefore argue that the apparent high-speed foraging style of Sowerby's beaked whales influences their dive duration and echolocation behaviour, and may enable access to a deep-sea prey population that is distinct from the one targeted by low-energy strategist beaked whales.

### Sprinter's anatomy

The higher energy strategy of Sowerby's beaked whales should be reflected in greater investment in organs compared with the extreme anatomical economy of the mesoplodonts studied to date (Pabst et al., 2016). However, our data suggest that Sowerby's beaked whales share some key anatomical specializations with their lower-energy cousins. Like Blainville's beaked whales, both tagged Sowerby's beaked whales descended passively in deep dives, indicating a net tissue density significantly greater than that of seawater (Biuw et al., 2003). Also comparable to Blainville's beaked whales is the distinctive stroke-and-glide gait with fast, high-acceleration strokes used in ascents from deep dives (Martín López et al., 2015). These strokes were proportionally shorter than those of Blainville's beaked whales, in keeping with the overall faster stroking in Sowerby's beaked whales. Martín López et al. (2015) suggest that this gait in Blainville's beaked whales may draw upon fast-twitch muscle fibres which curiously represent a large proportion of the swim muscle fibres in this species. The presence of a similar ascent gait in Sowerby's beaked whales suggests that they may have a similar muscle fibre profile, with a predominance of fast-twitch fibres serving both as an economical myoglobin reservoir (Velten et al., 2013) and as a source of thrust during deep-dive ascents.

### Reduced surface crypsis

A high versus low-energy regime place different constraints on behaviour. The slow-strategist beaked whales, including Blainville's, minimize time at the surface by performing a sequence of shallower, and silent, dives between deep foraging dives (Aguilar de Soto et al., 2020). Ascents from foraging dives are also performed at low pitch angles that can potentially translate animals horizontally a considerable distance (Martín-López et al., 2015). These seemingly inefficient behaviours both serve to reduce the risk of encounters with eavesdropping predators at the surface (Aguilar de Soto et al., 2020), suggesting a risk-averse behavioural syndrome. Both tagged Sowerby's beaked whales performed similar low-angle ascents from foraging dives. However, they spent the vast majority of the time between foraging dives at <20 m depth where they would be readily detectable by visual predators. During these prolonged near-surface intervals, tagged Sowerby's beaked whales engaged in fast horizontal travel with consistently high stroking rates. Stroking rates were especially high immediately after deep dives, and short bursts of extreme speed throughout the surface intervals, reaching up to 8 m s^−1^, suggest that they can make a much more energetic response to predators and competitors than their congener. Thus, the high-energy foraging strategy of Sowerby's beaked whales, and the increased metabolic rate that it implies, may also enable more robust options for predator defence. As the tag recordings include very little night data, we cannot test for potential diel changes in near-surface swimming behaviour, as has been reported in some locations for Blainville's beaked whales (Baird et al., 2008).

The occurrence of three separate close encounters with other clicking toothed whales in only 11 h of tag recording adds to the picture of a less risk-averse surface behaviour. These bouts of surface clicks could be produced by group members of the tagged individuals but this would imply that: (i) Sowerby's beaked whales, unlike Blainville's beaked whales, are prepared to risk detection near the surface by acoustic predators, and (ii) that they produce broadband clicks at shallow depths which are very different from their FM search clicks. Alternatively, the clicks could have been produced by the sympatric and locally abundant Risso's dolphin (Madsen et al., 2004; Visser et al., 2021a). Given the differentiation in diet and foraging depth between Risso's dolphins and Sowerby's beaked whales, these are unlikely to be direct food competitors (Visser et al., 2021a) and they are not known to associate (F.V., personal observation). Nonetheless, given the frequency of the encounters recorded by the tags, Risso's dolphins may sometimes actively seek out Sowerby's beaked whales or vice versa. In either case, this indicates that Sowerby's beaked whales are less cryptic at the surface and are able to withstand interference, and possibly predation, by high-speed swimming.

### Conclusions

Sowerby's beaked whales display a distinctly more energetic lifestyle than similar-sized Blainville's beaked whales, suggesting that the mesoplodonts exploit a diversity of energetic niches within the mesopelagic. We propose that the availability of these niches may have driven the development of matching sensory and locomotory strategies, helping to explain the adaptive radiation of the most speciose toothed whale family. The adoption of these suites of characteristics enables the division of prey resources by energetic strategy, with associated implications for competitive and risk-avoidance strategies. As data become available from more mesoplodonts, we predict that a diversity of behaviours, matched with varying energetic investment in organs, will be found in keeping with slow and fast lifestyles adapted to different deep-sea foraging niches.
